# Prescriptions and Dispensing

**Published:** 1907-10-19

**Authors:** 


### Therapeutics.

### PRESCRIPTIONS AND DISPENSING.

(*Continued from page* 18.)

### II. MEDICINES IN LIQUID FORM.

The medium in which a more active drug is dissolved or otherwise distributed is termed the *vehicle*; the vehicle may be itself inert, or it may have some subordinate medicinal property. In the majority of cases, in order that a drug which is taken internally may exercise its medicinal properties, it is necessary that it should be absorbed. If, then, the drug is already in solution in a liquid when taken into the body it will pass more readily and rapidly into the circulation than if it is in the solid state; a dissolved salt, for instance, taken into the stomach will more readily pass into the circulation than if the same salt were swallowed as a solid, requiring to be dissolved in the liquid contents of the stomach before absorption into the blood could begin. Generally, then, ready absorption and prompt exercise of activity are the desiderata which led to the liquid form of medicine being prescribed. Other considerations are sometimes of importance also, however; the insoluble bismuth salts, for example, are sometimes required to exercise a mechanical action in the stomach and intestines, forming a protective layer on the walls, and uniform distribution of such substances is better secured if they are already distributed evenly in a liquid before taking; in the case of a gargle or paint for the throat, or a rectal injection, proper application of the drug would be practically impossible without a liquid vehicle, and the same applies to liniments and lotions for external use.

The two chief aims in preparing medicines in the liquid form must be, first, to ensure that every ingredient shall be in such a condition that its full activity is unimpaired, or if its activity is necessarily lessened by some other ingredient, that such diminution shall be the least possible; and, secondly, to secure a perfectly even distribution of each ingredient throughout the whole of the medicine so that each dose shall contain the same proportion of the various constituents. The securing of these two objects is often not a perfectly simple matter, and the methods required in typical cases will have to be studied. It is important that the objects should be kept in mind, since they furnish the key to the methods to be used.

We will begin with "mixtures," or *misturæ*, by which name are designated medicines for internal use when consisting of more than one dose, taken in not very small quantity. A single dose ordered by itself is known as a "draught," or *haustus*, while concentrated mixtures to be taken in very small quantities are termed "drops" or *guttæ*. The methods of dispensing required in these cases are generally the same as for mixtures, and we shall for the most part deal with them together.

#### Simple Mixtures.

When a mixture consists solely of liquid ingredients which do not in any way decompose or combine with each other, very little is required beyond accurately measuring them and putting them into a bottle along with the vehicle. The following is an example of such a simple case:—

| | | |
|---|---|---|
| ℞ | Liquoris Ammonii Acetatis ... ... | ʒvj. |
| | Tincturæ Aurantii ... ... ... ... | ʒiij. |
| | Spiritûs Ammoniæ Aromatici ... ... | ʒij. |
| | Syrupi ... ... ... ... ... | ℥ss. |
| | Aquam ... ... ... ... | ad ℥vj. |

Even here, however, the order in which the ingredients are mixed is not without importance. The spirit of sal volatile should in this case be added after diluting the other preparations with most of the water; by so doing the loss of ammonia while filling up the bottle, and the darkening caused by its action on the colouring matter of the orange, are reduced to a minimum.

| | | |
|---|---|---|
| ℞ | Liquoris Bismuthi ... ... ... ... | ℥ss. |
| | Tincturæ Cardamomorum compositæ ... | ʒij. |
| | Tincturæ Gentianæ compositæ ... ... | ʒij. |
| | Acidi Hydrocyanici diluti ... ... ... | ♏xx. |
| | Aquam Menthæ piperitæ ... ... | ad ℥iij. |

In this case the hydrocyanic acid must be added last or there will be a very serious loss while filling up with the peppermint water. Mixtures containing hydrocyanic acid should always be labelled "Shake the Bottle." Vapour of hydrocyanic acid collects in the space above the liquid, especially when the bottle is partly emptied, and the shaking redissolves it and distributes it evenly through the mixture.

#### Solids in Mixtures.

If one of the ingredients in a mixture is a solid which is readily soluble in the vehicle the case is scarcely less simple than the preceding. In the following example—

| | | |
|---|---|---|
| ℞ | Potassii Bromidi ... ... ... ... | ʒij. |
| | Tincturæ Nucis Vomicæ ... ... ... | ʒj. |
| | Syrupi Limonis ... ... ... ... | ʒvj. |
| | Aquam ... ... ... ... ... | ad ℥ij. |

the bromide is easily soluble in a part of the water, and the liquids have then only to be mixed. There are, however, several points to be noted.

The usual rule when a solid is to be dissolved is to powder it in a mortar, preferably of glass, dissolve in part of the vehicle, and then strain the solution—if any foreign particles are visible in it—as often happens—through fine muslin into the bottle; in dealing with a salt like potassium bromide, which is very easily soluble, is usually quite clean, and is in large crystals from which any foreign particles can be readily removed, there is no objection to putting it straight into the bottle with some of the water, corking and shaking up till dissolved; but many soluble salts, if so treated, would cling about the neck of the bottle, some being lost very likely, and it is on the whole better to keep to the rule of dissolving before putting into the bottle. When it is necessary to shake up the bottle in the process of making a mixture it should always be corked, and not merely closed with the finger. The latter method is uncleanly, and entails loss of a small amount of the contents which clings to the finger.

(*To be continued.*)

